# Fixating targets in visual search: The role of dorsal and ventral attention networks in the processing of relevance and rarity

**DOI:** 10.1162/imag_a_00229

**Published:** 2024-07-17

**Authors:** Anja Ischebeck, Hannah Kreilinger, Joe Peiris Miller, Margit Höfler, Iain D. Gilchrist, Christof Körner

**Affiliations:** Department of Psychology, University of Graz, Graz, Austria; BioTechMed-Graz, Graz, Austria; Department for Dementia Research and Nursing Science, University for Continuing Education Krems, Krems, Austria; School of Psychological Science, University of Bristol, Bristol, United Kingdom

**Keywords:** fixation-related, fMRI, visual search, TPJ, rarity, relevance

## Abstract

The dorsal attention network, often observed to be activated in serial visual search tasks, has been associated with goal-directed attention, responsible for the processing of task relevance. In serial visual search, the moment of target detection constitutes not only a task-relevant event, but also a rare event. In the present fMRI experiment, we disentangled task relevance from item rarity using a fixation-based analysis approach. We used a multiple target search task, and participants had to report the number of targets among distractors in the display. We had also added rare distractors to the displays. We found that rare events (targets and rare distractors) activated the dorsal attention network more strongly than common distractors. More importantly, we observed that the left IPS and the left insula, belonging to the dorsal and ventral attention system, respectively, were more strongly activated for targets compared to rare distractors. Using multi-voxel pattern analysis, we found that activation in the TPJ, bilaterally, an area also associated with the ventral attention system, distinguished between target and rare distractor fixations. These results point to an expanded role of the TPJ that seems to process post-perceptual information which is linked to task relevance.

## 1 Introduction

Finding four-leaved clover is considered a good luck charm by some, perhaps because it is rare and difficult to spot among its three-leaved relatives. This is an example of a serial visual search task, because the desired object does not visually “pop out” and attention has to move from location to location until we find it (Treisman & Gelade, 1980). However, what happens when we finally detect the desired object, the target? Detecting a target is a task-relevant event because it typically entails some form of action, for example, memorizing the target location, or terminating the search, and/or initiating a manual response. Detecting a target is also a rare event because the visual environment typically contains many objects that are similar to the target but irrelevant (distractors). Investigating the processing of targets in serial visual search using fMRI can help identify brain areas that process task relevance. Although visual search tasks have been investigated several times using fMRI, studies investigating activation changes during the search process that distinguish between task-relevant objects and irrelevant objects are still rare (Ischebeck et al., 2021;Macaluso & Ogawa, 2018). In a precursor study, we found that targets activated the dorsal attention network more strongly than distractors (Ischebeck et al., 2021). In the present study, we ask whether task relevance is responsible for this difference and how the task relevance of a target is related to its rarity in the search environment.

It is important to distinguish task relevance from rarity. The detection of a search target is always a task-relevant event. However, in a typical search situation, it is also a rare event, as illustrated by the introductory example. Nonetheless, the visual environment may contain other, irrelevant objects (distractors) that are sometimes also rare. Thus, rarity is an incidental property of the target that may be shared by distractors. The defining property of the target, however, is its relevance. Because the detection and processing of task-relevant targets is often the basis for further interaction with the environment, the study of how the brain processes task relevance is of particular interest for understanding goal-directed behavior.

According to one of the most prevalent neuroscientific theories of attention (Corbetta & Shulman, 2002), attention is subserved by two separate networks of brain areas: one responsible for bottom-up attention, consisting of ventral fronto-parietal areas such as the temporo-parietal junction and the insula (or inferior frontal cortex), whereas the other is responsible for top-down attention, comprising, among others, the intraparietal sulcus and the frontal eye fields. Both networks are separable, for example, on the basis of low-frequency BOLD fluctuation patterns in resting-state fMRI studies (Fox et al., 2006;Yeo et al., 2011). It is also assumed that both attention systems interact. In the model by Corbetta and Shulman, the TPJ of the right hemisphere is assumed to serve as a “circuit-breaker” that alerts the top-down attention network to salient and previously unattended stimuli. The hemispheric asymmetry of the networks was suggested on the basis of observations from neglect patients where right hemispheric lesions of the TPJ have more persisting consequences than left hemispheric lesions (Corbetta & Shulman, 2011). However, the role of the TPJ may not be limited to bottom-up stimulus-driven processing. The TPJ of the left hemisphere also seems to be involved in attentional control (Geng & Vossel, 2013) and it may reflect more high-level processes that occur post-perceptually, such as contextual updating and adjustments of top-down expectations, in other words, processes that relate to the relevance of the stimulus (see alsoVossel et al., 2014).

Shulman et al. (2007)have also suggested an additional role of the right TPJ in the processing of target relevance. Using a combined motion detection and search task, they found that distractor processing was associated with a deactivation of the rTPJ. Higher deactivation of the rTPJ was also related to better detection of subsequent targets. As this deactivation preceded target detection, the deactivation of the rTPJ might be related to more than post-detection processes, such as contextual updating. Shulman et al. suggested that the rTPJ plays a role in filtering out irrelevant information to prevent inappropriate attention shifts. These results indicate that the TPJ plays a role in focused attention and relevance processing that goes beyond the traditional view of being involved in reorienting attention to salient stimuli.

In the present study, we aimed to disentangle task relevance from rarity in target processing. We used a multiple target search task (e.g.,Körner & Gilchrist, 2008). Participants’ eye movements were tracked while they searched for and reported the number of targets among distractors in the display. We also added rare distractors to the displays which were to be ignored. To identify brain areas associated with task relevance rather than item rarity, we compared activation arising from the fixation of targets with activation arising from fixating rare distractors. Importantly, we designed displays in which targets and distractors shared visual features and items could only be identified by direct foveation. All items in the display were equally salient. Peripheral vision could not provide any information about objects that were likely to be the target, and thus, peripheral vision could not contribute to the guidance of the search process (Körner & Gilchrist, 2007). This particular setup makes it unlikely that the ventral attention system was involved in our task in its traditional role of orienting attention in a stimulus-driven manner. In this way, the design of our stimuli allowed us to assess more clearly whether activation in areas usually associated with the ventral attention system have a role that is different from the bottom-up guidance of attention.

We used target and distractor fixations as events of interest in a fixation-based event-related analysis. This type of analysis has been used successfully before to explore brain activation, for example, during reading (Carter et al., 2019;Henderson et al., 2015,^2016^;Richlan et al., 2014;Schuster et al., 2016), natural scene or object viewing (Henderson & Choi, 2015;Henderson et al., 2020;Kuniecki et al., 2017;Marsman et al., 2012,^2016^), and overt serial visual search (Ischebeck et al., 2021;Macaluso & Ogawa, 2018). We used standard GLM as well as multivariate pattern analysis to compare brain activation patterns between targets, rare distractors and common distractors.

Visual search tasks have been investigated before. When more difficult search tasks (e.g., serial visual search) are compared to easier search tasks (e.g., pop-out or parallel search), typically activations in areas of the dorsal attention system are observed (Donner et al., 2000,^2002^;Fairhall et al., 2009;Kim et al., 2012;Leonards et al., 2000). This was interpreted as being due to the goal-directed attention required in the more difficult search tasks. There is one fMRI study byMacaluso and Ogawa (2018)that also employed a fixation-based analysis approach. They asked their participants to navigate through a 3D search display with targets and different distractors and compared fixations on targets with fixations on different distractor types. They found stronger activations for targets than for distractors in the right inferior parietal lobe, an area that belongs to the dorsal attention system.

For the present experiment, we thus expect targets to involve goal-directed attention because they are task-relevant objects. More precisely, an activation of the dorsal attention network should be expected for targets as compared to distractors. Indeed, in one of our own studies where we had participants search for a small number of targets (“T”s) among numerous distractors (all “L”s), we found that targets activated the dorsal attention network more strongly than distractors (Ischebeck et al., 2021). In that particular study, however, the observed activation of the dorsal attention network could have been due to the task relevance of targets as well as their relative rarity. In the present study we added rare distractors to the display in an effort to disentangle target processing from item rarity.

Apart from the dorsal attention network, we also assume that areas usually assigned to the ventral attention system might additionally contribute to the processing of task relevance. Areas of the ventral attention system such as the TPJ have been implied with target processing in the Posner cueing paradigm (Doricchi et al., 2010;Jurewicz et al., 2020;Rosen et al., 1999;Vossel et al., 2006,^2009^) as well as in oddball studies (Horovitz et al., 2002;Kiehl et al., 2005;Linden et al., 1999; for a meta-analysis, seeKim, 2014). It may not seem obvious to associate visual search with oddball tasks but the stream of events in our visual search task—serialized by saccadic eye movements—can be compared to a fast oddball task, where rare events such as targets and novels are presented among frequent distractors or standards. As in our paradigm there was no possibility to peripherally identify items during search, participants could not anticipate when they were going to find a target in the sequence of fixations during search. For the present experiment, we therefore hypothesized that areas of the ventral attention network might become activated alongside areas of the dorsal attention system for targets compared to rare distractors.

## 2 Methods

### 2.1 Participants

Participants were screened to verify their usability for combined eye tracking and fMRI measurements. For a participant to be considered for the main experiment, the eye tracker had to be calibrated with a spatial resolution of better than 0.50° v.a. with the participant in the scanner. During screening, participants completed a single training run of 28 trials, to familiarize themselves with the task. From 25 screened adult participants, 21 (13 females, 8 males) were classified as trackable and were invited to participate in the experimental session one week after the screening. Eighteen participants were right-handed. All participants had error rates of below 20%, no history of neurological or psychiatric disease, and normal or corrected-to-normal vision (contact lenses). Mean age of the participants was 24.0 years (*SD*= 1.97, range 20–28 years).

Participants were either paid €35 or received course credit for their participation. Written informed consent was obtained from all participants prior to the experiment. The study was approved by the ethics committee of the University Graz and was conducted according to the general principles of the Declaration of Helsinki.

### 2.2 Design and stimuli

Participants completed a series of searches for multiple identical targets in displays among distractors and had to report the number of targets in the display (0, 1, or 2). The experiment used a 2 × 2 design with the two factors target (zero targets and one target, 0T, 1T) and rare distractor (zero and one rare distractor, 0RD, 1RD) yielding four experimental search conditions in total: 0T_0RD, 0T_1RD, 1T_0RD, and 1T_1RD. To ensure an exhaustive search in the four search conditions of interest, there were also two catch trial conditions with two targets, 2T_0RD and 2T_1RD.

Each display consisted of a total of 30 items: targets (“T”s), common distractors (“L”s), and rare distractors (upside-down “L”s). For 10 of the 21 participants, the identities of target and rare distractor were switched in order to control for differences in the visual appearance of the stimuli. Each item in the display was presented in white on a black background and surrounded by a white circle (Körner & Gilchrist, 2007), in order to prevent letters from being peripherally identifiable (Bouma, 1970) and to present a clear saccade target. Letters subtended a viewing angle of 0.14° at a viewing distance of 127 cm. Circle diameter was 0.45° v.a. The items appeared randomly at the intersections of an imaginary 7 x 7 grid with a random deviation of maximally ± 0.16° in horizontal and vertical direction. The minimum inter-item distance was 1.14°. The viewing angle of the whole display subtended an area of 10.21° x 10.21°.

**Fig. 1.**
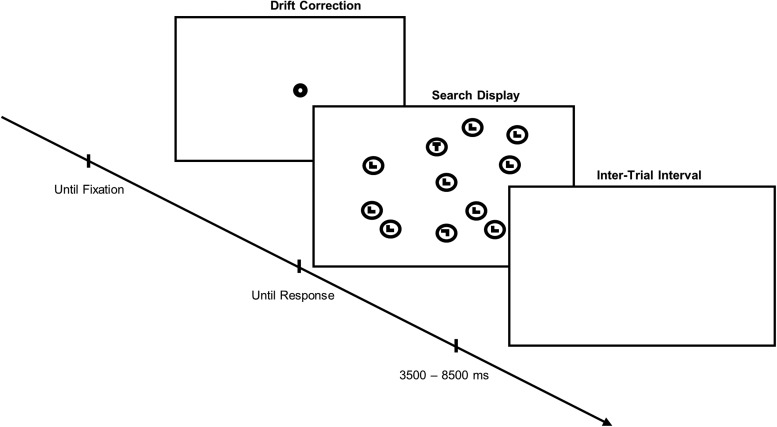
Trial timing. After drift correction, the search display is presented (30 items in total). Common distractors are “L”s. “T”s, and upside-down “L”s can be targets and rare distractors, depending on the instruction. Participants are instructed to indicate the number of targets (zero, one, or two) by pressing one of three buttons on a button box. The search display is then replaced by a fixation disk for the duration of the inter-trial interval. In the experiment, stimuli were presented in white on a black background. The search display shown here is symbolic and not drawn to scale.

Participants reported on the number of targets present by pressing one of three buttons corresponding to 0, 1, or 2 targets found. We simultaneously recorded manual responses, eye movements, and fMRI data (Fig. 1).

### 2.3Task and procedure

The task was to search each display and report the number of targets present, by pressing one of three buttons on a four-button response pad using the dominant hand. Response buttons were ordered for 0 to 2 targets from left to right, with one finger (from index to ring finger) kept on each button. Participants were instructed to respond as quickly and as accurately as possible.

Each trial started with a drift correction. A fixation disc was presented in the center of the screen until fixation was manually registered by the experimenter. After drift correction, the search display was presented until the manual response by the participant terminated the trial. Between trials, there was a variable inter-trial interval (between 3500 and 8500 ms, in 500 ms steps) during which a fixation cross was presented. The variable duration of the inter-trial interval was chosen to improve the estimability of the hemodynamic response (Liu et al., 2001).

The experiment consisted of six runs with 28 trials each, yielding a total of 168 trials. Of these 28 trials, there were 6 for each of the four exhaustive search conditions (0T_0RD, 1T_0RD, 0T_1RD, 1T_1RD) plus two trials for each catch trial condition (2T_0RD, 2T_1RD). The eye tracker was calibrated between runs.

Participants lay supine in the scanner with foam paddings to stabilize the head, and earplugs to protect them from the scanner noise. Prior to the start of the first session, an anatomical scan of the participant was acquired. The scanner was started separately for each run. The functional measurements lasted for approximately 60 minutes, with a 5 minute break after the third run. During the break, participants remained in the scanner.

### 2.4 MR recording

Functional and anatomical data were collected using a Siemens 3 Tesla Skyra MR scanner. For the functional measurement, a multiband accelerated EPI pulse sequence developed by the University of Minnesota (e.g.,Xu et al., 2013) was used. Fifty-two slices were acquired with a slice thickness of 2.5 mm, 0.25 mm gap, and in-plane resolution 2.5 x 2.5 mm (TR = 1250 ms, TE = 40 ms, flip angle = 60°, FOV = 240 mm). For the anatomical measurement, a T1 MPRAGE sequence with an in-plane resolution of 1 x 1 mm and a slice thickness of 1 mm, 0.5 mm gap (TR = 1560 ms, TE = 2.07 ms, flip angle = 9°, FOV = 256 mm) was employed.

### 2.5 Eye movement recording and synchronization with MR recording

Eye tracking data were recorded during the fMRI measurement. An Eyelink 1000 eye tracking system with an infrared camera and a 50 mm lens (SR Research, Ontario, Canada) was used to record two-dimensional eye movements. A 32-inch LCD monitor (Nordic Neuro Lab, Bergen, Norway) with 1920 × 1080 pixel resolution and a vertical refresh rate of 60 Hz was used for display presentation. It was positioned behind the bore of the scanner. The displays were viewed in the MRI scanner using an MR- and infrared-compatible mirror mounted onto the head coil and tilted at 45° so that the participant was able to view the entire display. The distance from the eye to the monitor was ~127 cm.

Custom-written software in C++ (Microsoft Visual Studio 2007), which also used functions from the EyeLink toolbox provided by SR Research, was used to control stimulus presentation, to record manual responses and eye movements, and to accomplish the synchronization with the MR scanner. Prior to the scanning of each run, we used a standard 9-point calibration procedure, in which participants fixated on 9 circles which appeared one after the other on screen covering the entire display area. During validation, another series of circles were presented at the same locations in order to validate the accuracy of the calibration. Data were recorded from the dominant eye at a sampling rate of 500 Hz.

The eye tracker was mounted on a wooden frame attached to the stand of the LCD display monitor, and was connected via a fiber-optic cable to a dedicated Host PC running EyeLink software. The Host PC was connected via an Ethernet link to a dedicated Display PC controlling the experimental display. The Display PC received input via USB from a Current Designs 932 interface (Current Designs, Philadelphia, USA). This interface was connected to a Current Designs 8 Button Bimanual Straight Lines response device via an optical port and to the MR scanner, from which it received an optical trigger signal at the start of the first and last functional volume of each run. This trigger signal was passed on via the Display PC to the Host PC for offline synchronization of eye tracking and fMRI data.

### 2.6 Data analysis

#### 2.6.1 Eye movement analysis

Fixations were defined as the periods between saccades, and were detected using the default SR research algorithm. Each fixation was assigned to the item that was within the shortest Euclidean distance of the fixation point. Saccades were detected if the velocity of the eye’s position surpassed a threshold of 30°/s, with a minimum acceleration threshold of 9500°/s^2^.

#### 2.6.2 Fixation preprocessing

When an item was refixated immediately, that is without saccades to other items in between, these immediate refixations were treated as a single fixation (collapsed fixation). The fixation duration of the immediate refixations was added to the fixation duration of the first fixation. The onset time of the first fixation was taken as the onset of the collapsed fixation. Direction and amplitude of the preceding saccade were calculated for the first collapsed fixation, while direction and amplitude of the following saccade were calculated from the last collapsed fixation. We then determined the serial position of each fixation within a trial (fixation rank).

#### 2.6.3 Fixation rank matching

We conducted fixation rank matching only for three exhaustive search conditions with events of interest (1T_0RD, 0T_1RD, 1T_1RD, seeTable 1). We did not match fixation events from the catch trials (2T_0RD, 2T_1RD). We had added only two catch trials each per block and these searches were also non-exhaustive, that is, the search could be aborted once the second target was found. We also did not use fixations from the 0RD_0T condition. Our main goal was to match exactly one common distractor fixation to one target or rare distractor, respectively, while keeping all other influencing factors, such as potential differences between the conditions, constant. Finally, we also did not match fixation events in the case of error trials. The most common errors in serial visual search are misses, that is, a target has not been detected. We reasoned that, in these cases, targets were processed more similarly to common distractors.

**Table 1.** Calculated contrasts, matched and matching fixation events, and search conditions used in the contrast.

Contrast	Matched fixation	Matching fixation	Search conditions used
Target vs. common distractor	T	CD_T	1T_0RD, 1T_1RD
Rare distractor vs. common distractor	RD	CD_RD	0T_1RD, 1T_1RD
Target vs. rare distractor	nm		1T_0RD, 0T_1RD, 1T_1RD
Common distractor (targets) vs. Common distractor (rare distractor)	nm		1T_0RD, 0T_1RD, 1T_1RD

*Note.*T = target, RD = rare distractor, CD = common distractor, CD_T = common distractor rank-matched to T, CD_RD = common distractor rank-matched to RD, nm = not matched; see Data Analysis section for a description of the matching procedure. Targets were not matched to rare distractors in the T vs. RD contrast. The CD_T vs. CD_RD contrast was calculated to test for possible confounds in the T vs. RD contrast due to differences in fixation ranks. The 1T_1RD condition was not used in the MVPA.

For each trial, we matched only first fixations on an item of interest (target or rare distractor). We first determined the fixation rank of the first fixation of a target T or rare distractor RD, respectively. The fixation rank is an item’s ordinal position in the temporal sequence of fixations. For a fixation event of interest (T or RD), we selected a common distractor fixation with the same fixation rank from one of the other trials. For example, we matched a target fixation T to a common distractor fixation (CD_T) with the same fixation rank.

Matching was conducted separately for each run (28 trials). We first randomized the order of the trials. This was done to prevent the matching procedure to pick distractors preferentially from early trials of a run. A target or rare distractor with a certain fixation rank was matched with a common distractor with the same fixation rank but from a different trial of the same condition. This common distractor was then excluded from the matching procedure for the following target and rare distractor fixations so that it could not be selected again. We only considered exact matches, leaving out target and rare distractor fixations for which no common distractors with the exact fixation rank could be found. This resulted in a loss of 118 target fixations out of a total of 1477 (7.99 %), and a loss of 91 rare distractor fixations out of a total of 1394 (6.53 %). The loss per subject ranged from 0 % to 25.4 % for target fixations and from 0 % to 18.3 % for rare distractor fixations. In the 0T_1RD condition, 97.48 % of all rare distractor fixations were matched successfully. In the 1T_0RD condition, 91.57 % of all target fixations were matched successfully. In the 1T_1RD condition, 89.72 % of rare distractor and 92.43 % of target fixations were successfully matched. For all conditions, the matching procedure yielded as many common distractor events for analysis as there were target and rare distractor events.

#### 2.6.4MRI data analysis

All data were preprocessed using SPM 12 (Wellcome Department of Cognitive Neurology, London, UK) and Matlab R2015b. The model contained all six runs, modeled as separate sessions. The first two functional images from each run were discarded for signal stabilization. We calculated two analyses, a standard analysis based on the canonical form of the BOLD response and the general linear model (GLM) as implemented in SPM12, and a whole-brain multi voxel pattern analysis (MVPA) using a searchlight approach.

For the standard analysis, we used functional images that were motion corrected, normalized, and smoothed with a 6 mm FWHM Gaussian kernel. For normalization, we first coregistered the anatomical image to the mean image of the functional runs of an individual. The anatomical image was then segmented using 6 tissue maps and normalized, generating forward deformation field parameters for normalization. The parameters from the normalization of the anatomical image were then used to normalize the functionals. For the first (standard) analysis, we calculated a general linear model using fixations as events of interest. Autocorrelations were modeled with AR(1). We modeled the BOLD response with two regressors for target fixations (from the 1T_0RD and 1T_1RD search conditions), two regressors for rare distractors (from the 0T_1RD and 1T_1RD search conditions), and their separately fixation rank-matched common distractors from these conditions, yielding eight regressors in total. Fixation events from the 0T_0RD search condition, from the catch trials (2T_0RD and 2T_1RD), as well as fixation events from error trials were not modeled. These trials are hence included in the implicit baseline. Because it is possible that including these trials as a regressor of no interest might make the analysis more sensitive, we also ran a control analysis, modeling the hemodynamic response function (hrf) with the trial onsets and durations (reaction time). The results of the control analysis were very similar to the analysis reported here. In the group analysis of our main contrast of interest (targets vs. rare distractors), the two clusters in the left IPS and the left insula remained significant, but were a bit smaller, compared to the analysis results reported here. We therefore decided to report the original analysis here, modeling only the fixation events. The BOLD response for the fixation events was modeled with a duration of 0 using the canonical form of the hrf. A high-pass filter (cut-off frequency: 1/128 Hz) was used to remove low-frequency drifts. The six motion parameters from the realignment procedure per run were also entered as parameters of no interest. No global normalization was used. We calculated the following contrasts: First, targets (T) versus fixation rank-matched common distractors (CD_T). Second, rare distractors (RD) versus fixation rank-matched common distractors (CD_RD). Then, we compared target fixations (T) with rare distractor fixations (RD). In this contrast, target fixations were not fixation rank-matched to rare distractor fixations. To test for possible activation differences induced by fixation rank differences between both conditions, we also compared their fixation rank-matched common distractors (CD_T and CD_RD). They had the same fixation rank as the targets and distractors. If there was a confound due to different fixation ranks of targets and rare distractors, it should also appear in this contrast. A second-level analysis (one-sample*t*-test) was conducted using the contrasts calculated on the single-subject level. We also calculated percent signal change in the two activated clusters from the contrast targets (T) versus rare distractors (RD) to illustrate the activity in all four conditions. For data extraction we used the Marsbar toolbox (http://marsbar.sourceforge.net).

For the MVPA we used functional images that were only motion corrected to preserve the spatial resolution of the fMRI data. Different from the standard analysis, which investigates activation amplitude differences in specific areas on the basis of spatially smoothed images, MVPA has the potential to separate activation patterns of targets and rare distractors by exploiting the activity in neighboring voxels using unsmoothed data. The MVPA was calculated on the basis of the beta-images from an analysis based on the same general linear model (GLM) as used for the standard analysis. We used a searchlight approach (Kriegeskorte et al., 2006), analyzing the activation pattern of small spherical clusters with a radius of 4 voxels at each position in the brain. This approach enables a whole-brain analysis that does not rely on predefined regions of interest. The classifier was trained on five of the six runs per participant. The data from the sixth run was then used to test the generalization of the classification procedure. The procedure was repeated until every run had been used as test run (leave one run out cross-validation procedure). This analysis yields an accuracy map for each participant with prediction accuracies averaged over the six test steps. We used the TDT Toolbox (Hebart et al., 2015) for the MVPA. For each subject, classification accuracy maps were calculated for the comparison of targets T versus rare distractors RD from the 1T_0RD and the 0T_1RD conditions, respectively. The accuracy maps were then normalized based on the normalization parameters of the anatomical image. Finally, the normalized accuracy maps were smoothed using a 6 mm FWHM Gaussian kernel. A second-level analysis (one-sample*t*-test) was conducted on the normalized and smoothed individual accuracy maps using SPM 12. We calculated the average classification accuracies for all significant clusters in the contrast targets versus rare distractors. For all analyses, we used a threshold with an initial uncorrected*p*-value of less than 0.001, reporting only clusters that survived FWE correction on cluster level (*p*< 0.05). The labels of the activation peaks were based on the automatic anatomic labeling (aal) toolbox (Tzourio-Mazoyer et al., 2002).

## 3 Results

### 3.1 Error rates and response times

Due to technical errors, 17 trials out of a total of 3528 (0.48 %) had to be excluded. Another 199 trials of the remaining 3211 trials (6.20 %) had to be discarded due to errors. Error rates and mean response times (RTs) per condition are given inTable 2. For the following ANOVAs, we only considered trials with 0 or 1 targets or rare distractors, respectively (conditions 0T_0RD, 0T_1RD, 1T_0RD, and 1T_1RD) because these constitute exhaustive searches. In the catch trials with two targets (conditions 2T_0RD and 2T_1RD), the search could terminate once a second target was found.

**Table 2.** Mean behavioral measures (and SDs) of subject performance.

Search condition	Error rate (%)	Response time (s)	Number of fixations
0T_0RD	0.66 (1.50)	10.806 (1.656)	25.54 (1.60)
0T_1RD	0.79 (1.99)	11.258 (1.967)	25.91 (1.90)
1T_0RD	8.07 (6.96)	10.974 (1.787)	25.67 (2.21)
1T_1RD	8.24 (5.53)	11.289 (1.971)	25.74 (2.25)
2T_0RD	12.45 (11.44)	9.018 (2.357)	20.71 (3.29)
2T_1RD	13.56 (9.64)	8.879 (1.916)	20.20 (2.74)

A 2 × 2 repeated-measures ANOVA for the error rates with the factors target (0, 1) and rare distractor (0, 1) showed a significant main effect for target (*F*(1,20) = 32.79,*MSE*< 0.01,*p*< 0.001,$\eta_{p}^{2}$= 0.62). The participants made more errors when a target was present (*M*= 8.16 %,*SD*= 6.29 %) than when it was absent (*M*= 0.73 %,*SD*= 1.76 %). This higher error rate was due to missed targets. It is a standard result in visual search that participants commit more errors by missing targets than by producing false alarms (Zenger & Fahle, 1997). Rare distractors did not increase error rates, as there was no significant effect for the factor rare distractor, (*F*(1,20) = 0.07,*MSE*< 0.01,*p*= 0.80; the interaction was also not significant,*F*(1,20) = 0.001,*MSE*< 0.01,*p = *0.98).

Only RTs from correctly answered trials were analyzed further. As before, we used a 2 × 2 ANOVA to analyze RTs. RTs did not differ between conditions with a target present (*M*= 11.13 s,*SD*= 1.88 s) compared to conditions without a target (*M*= 11.03 s,*SD*= 1.82 s,*F*(1,20) = 0.46,*MSE = *0.45,*p = *0.51). This was expected, as conditions with 0 or 1 target require an exhaustive search of the display. It is possible that participants rechecked rare items in the display so that their presence in the display might slow down response times. Indeed, trials with a rare distractor were completed more slowly (*M*= 11.27 s,*SD*= 1.97 s) than trials without a rare distractor (*M*= 10.89 s,*SD*= 1.72 s), which was reflected in a significant main effect (*F*(1, 20) = 14.76,*MSE*= 0.21,*p*= 0.001,$\eta_{p}^{2}$= 0.43). The interaction between target and rare distractor was not significant (*F*(1,20) = 2.01,*MSE*= 0.05,*p*= 0.17). RTs in trials with two targets were shorter (*M*= 8.95 s,*SD*= 2.07 s) than in trials with 0 or 1 targets (*M*= 11.08 s,*SD*= 1.80 s,*t*(20) = 9.38,*p*< 0.001,*d*= 2.05). This was expected because trials with two targets constituted self-terminating searches.

### 3.2 Eye movements

For all eye movement analyses, only data from correct trials were considered. For the ANOVA, we again only considered the trials with 0 or 1 targets (conditions 0T_0RD, 0T_1RD, 1T_0RD, and 1T_1RD). We conducted a 2 × 2 repeated-measures ANOVA for the average number of fixations with the factors target (0, 1) and rare distractor (0, 1). Similar to the ANOVA results for the RTs, there was no difference between trials with a target (*M*= 25.71,*SD*= 2.23), compared to trials without one (*M*= 25.72,*SD*= 1.75),*F*(1,20) = 0.007,*MSE*= 0.82,*p = *0.94 (seeTable 2). There was, however, a greater number of fixations for trials with a rare distractor (*M*= 25.83,*SD*= 2.08) compared to trials without one (*M*= 25.60,*SD*= 1.93). This led to a significant main effect of the factor rare distractor,*F*(1, 20) = 4.46,*MSE*= 0.23,*p*= 0.047,$\eta_{p}^{2}$= 0.18. The interaction between target and rare distractor was not significant,*F*(1,20) = 2.59,*MSE*= 0.19,*p = *0.12). In trials with two targets, there were fewer fixations (*M*= 20.5,*SD*= 2.81) than in trials with 0 or 1 target (*M*= 25.7,*SD*= 1.93;*t*(20) = 15.8,*p*< 0.001,*d*= 3.44). Again, this was due to the self-terminating search in the trials with 2 targets. Thus, the pattern of results for the number of fixations matched the pattern obtained for the manual RTs.

To check whether fixation durations were longer for rare items in the display, we conducted a one-way repeated-measures ANOVA with the factor item type. This factor had four levels: target (T), rare distractor (RD), fixation rank-matched distractors for targets (CD_T), and fixation rank-matched distractors for rare distractors (CD_RD). We analyzed fixation durations from the conditions 0T_1RD, 1T_0RD, and 1T_1RD. This analysis yielded a significant effect of item type,*F*(2.04,40.78) = 16.92,*MSE*= 2908.0,*p < *0.001,$\eta_{p}^{2}$= 0.46 (degrees of freedom were Greenhouse-Geisser corrected). Specifically, Newman-Keuls post-hoc tests showed that target fixations lasted longer than any other fixation types, and RD fixations lasted longer than fixation rank-matched common distractor fixations for targets (CD_T) and rare distractors (CD_RD), all*p*s < 0.05 (seeTable 3).

**Table 3.** Mean fixation duration and saccade amplitudes (and SDs) for targets (T) and rare distractors (RD) and their fixation rank-matched common distractors CD_T and CD_RD.

Item type	Fixation duration (ms)	Preceding saccade amplitude (deg v.a.)	Following saccade amplitude (deg v.a.)
T	465 (120)	3.54° (0.72°)	3.50° (0.70°)
RD	430 (123)	3.48° (0.64°)	3.55° (0.86°)
CD_T	368 (77)	3.53° (0.60°)	3.53° (0.70°)
CD_RD	367 (74)	3.56° (0.68°)	3.46° (0.67°)

It is basically possible that the difference in RTs and fixation durations between targets and rare distractors might influence activation differences between targets and rare distractor fixations. We, therefore, conducted a control analysis where we added regressors for RT and fixation duration. In the results, there was no significant activation explained by these regressors. This is similar to the results of our previous study (Ischebeck et al., 2021) where we had added RT and fixation duration as an additional regressors into the analysis but had found no significant modulation of activation patterns.

Next, we wanted to make sure that differences in the activations for targets and distractors were not due to differences in saccade lengths. To analyze whether item type influenced the preceding and following saccade amplitudes, we calculated a one-way repeated-measures ANOVA with the factor item type as before. These analyses showed no statistically significant differences for the factor item type. The average saccade amplitude following a fixation was 3.31° v.a. (*SD*= 0.72),*F*(3,60) = 0.29,*MSE*= 0.109,*p*= 0.83, and the average amplitude preceding a fixation was 3.52° v.a. (*SD*= 0.65),*F*(3,60) = 0.25,*MSE*= 0.097,*p*= 0.86. Averages and standard deviations for the different conditions are given inTable 3.

### 3.3 Imaging data

**Fig. 2.**
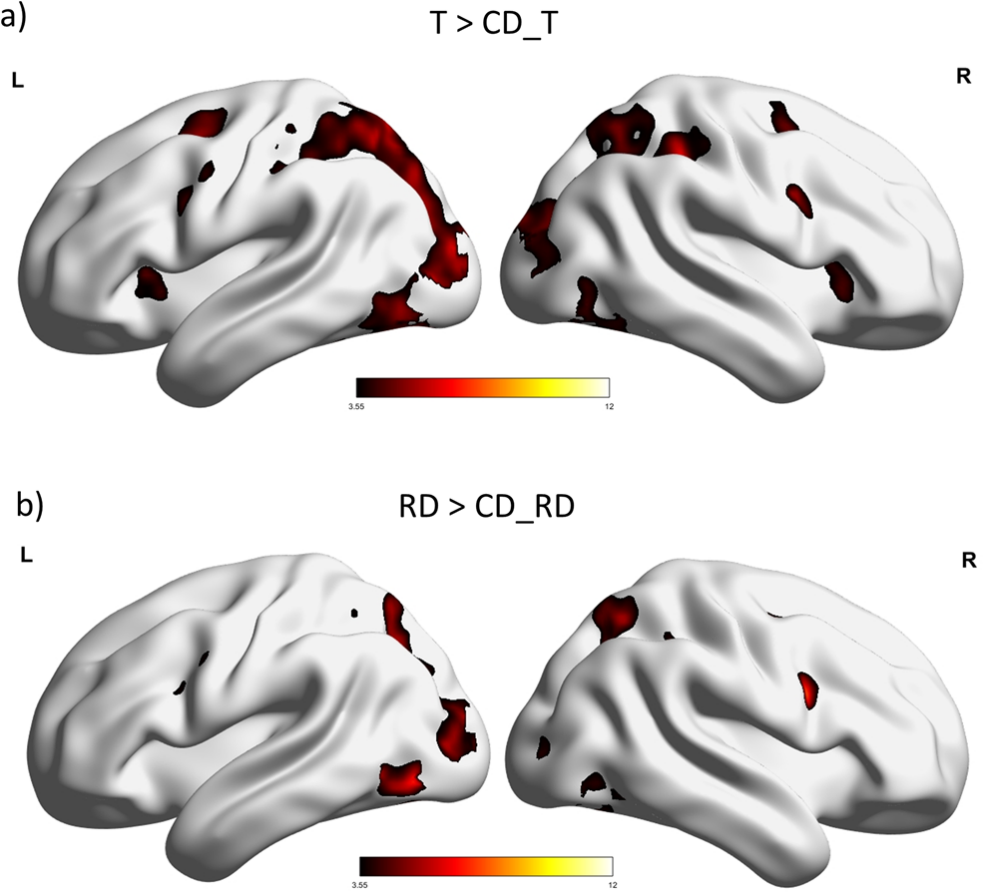
Results of the standard GLM analysis: (a) targets versus common distractors (T > CD_T) (b) rare distractors > common distractors (RD > CD_RD) threshold:*p*< 0.001 uncorrected, showing only clusters with*p*< 0.05 FWE corrected on cluster level. T = targets, CD_T = common distractors, fixation rank-matched to the targets, RD = rare distractors, CD_RD = common distractors, fixation rank-matched to the rare distractors. Results are visualized with BrainNet Viewer (http://www.nitrc.org/projects/bnv/) byXia et al. (2013).

First, we investigated the processing of rarity by comparing general activation differences between fixations on rare events such as targets and rare distractors with fixations on common distractors (“Ls”). To compare target fixations with fixation rank-matched common distractor fixations (T > CD_T), we used fixations in the 1T_0RD and 1T_1RD condition, that is, displays with 1 target and 0 or 1 rare distractor (seeTable 1). Similar to earlier findings (Ischebeck et al., 2021), we found significantly stronger activations for target fixations compared to fixation rank-matched common distractor fixations in a wide range of areas in the visual cortex and in fronto-parietal brain areas. Specifically, activations were observed in the superior and inferior parietal lobule, bilaterally and the left inferior occipital gyrus. In the frontal cortex, the precentral sulcus, the insulae, bilaterally, the supplementary motor area, and the right inferior frontal gyrus were activated (seeFig. 2a, andTable 4for the results). In the reverse contrast (CD_T > T), we observed significant clusters in the left anterior cingulate gyrus (coordinates: -2 28 14, k = 1855, Z = 5.17), the left posterior cingulate gyrus (coordinates: -4 -52 30, k = 579, Z = 5.05), and the left angular gyrus (coordinates: -48 -72 32, k = 138, Z = 3.93). The clusters disappeared when the results were masked with task activation (all conditions vs. implicit baseline), indicating that these areas are part of the default mode network, that is, they were deactivated during the task rather than activated. To compare the activation for rare distractors with activation for fixation rank-matched common distractors (RD > CD_RD), we used fixations in the 0T_1RD and 1T_1RD condition, that is, displays with 1 rare distractor and with 0 or 1 target. We observed stronger activations for rare distractors in the visual cortex and the parietal lobe. Activations were observed in the left middle occipital gyrus, the left and right fusiform gyrus, and the left superior parietal lobule (Fig. 2b,Table 4). In the reverse contrast (CD_RD > RD), we observed significant clusters in the left anterior cingulate gyrus (coordinates: -10 34 -2, k = 447, Z = 4.65), the left superior frontal gyrus (coordinates: -22 36 44, k = 134, Z = 4.21), and the left angular gyrus (coordinates: -52 -66 34, k = 169, Z = 3.93). These clusters also disappeared when the results were masked with task activation (all conditions vs. implicit baseline). To assess the commonalities between both activation patterns (T vs. CD_T and RD vs. CD_RD), we also calculated a conjunction analysis. However, we refrained from reporting its results here for the sake of brevity and because we were mainly interested in the contrast indicating task relevance (T vs. RD).

**Table 4.** Results of the standard GLM analysis: comparisons of target fixations and rare distractor fixations with common distractor fixations.

Hemisphere	Region	x	y	z	k	Z
	T > CD_T					
Left	Inferior occipital gyrus	-40	-80	-10	12280	5.62
Left	Superior parietal lobule	-18	-66	52	12280*	5.58
Right	Angular gyrus	32	-64	42	12280 *	5.39
Right	Inferior parietal lobule	42	-36	50	12280 *	4.98
Left	Cerebellum	-4	-74	-22	12280 *	4.76
Right	Supplementary motor area	4	14	50	1531	5.52
Right	Insula	34	22	-2	304	4.86
Left	Insula	-28	20	4	429	4.73
Left	Precentral gyrus	-42	0	30	432	4.72
Right	Precentral gyrus	42	6	28	326	4.72
Right	Thalamus	24	-24	-4	183	4.58
Left	Cerebellum	0	-54	-34	130	4.55
Right	Inferior frontal gyrus	44	28	30	305	4.54
Right	Lingual gyrus	6	-30	-4	124	4.47
Right	Pallidum	14	0	4	161	4.24
	RD > CD_RD					
Left	Fusiform gyrus	-36	-72	-14	6146	5.11
Left	Middle occipital gyrus	-14	-94	0	6146 *	5.05
Right	Calcarine gyrus	8	-90	6	6146 *	5.03
Right	Fusiform gyrus	32	-74	-6	6146 *	4.87
Left	Superior parietal lobule	-22	-66	54	160	4.16
	T > RD					
Left	Intraparietal sulcus	-34	-46	44	201	4.43
Left	Insula	-28	18	-4	174	4.10
Left	Middle frontal gyrus	-26	6	52	134	4.06

*Note.*SeeTable 1for the search conditions used in each contrast. Statistical parameter maps were thresholded with an initial threshold of*p*< 0.001 uncorrected, reporting only clusters that survived FWE correction on cluster level (*p*< 0.05). * Activation is part of a bigger cluster. Coordinates are reported as given by SPM12 (MNI space). k = cluster size, Z = Z value for the maximally activated voxel of the cluster.

**Fig. 3.**
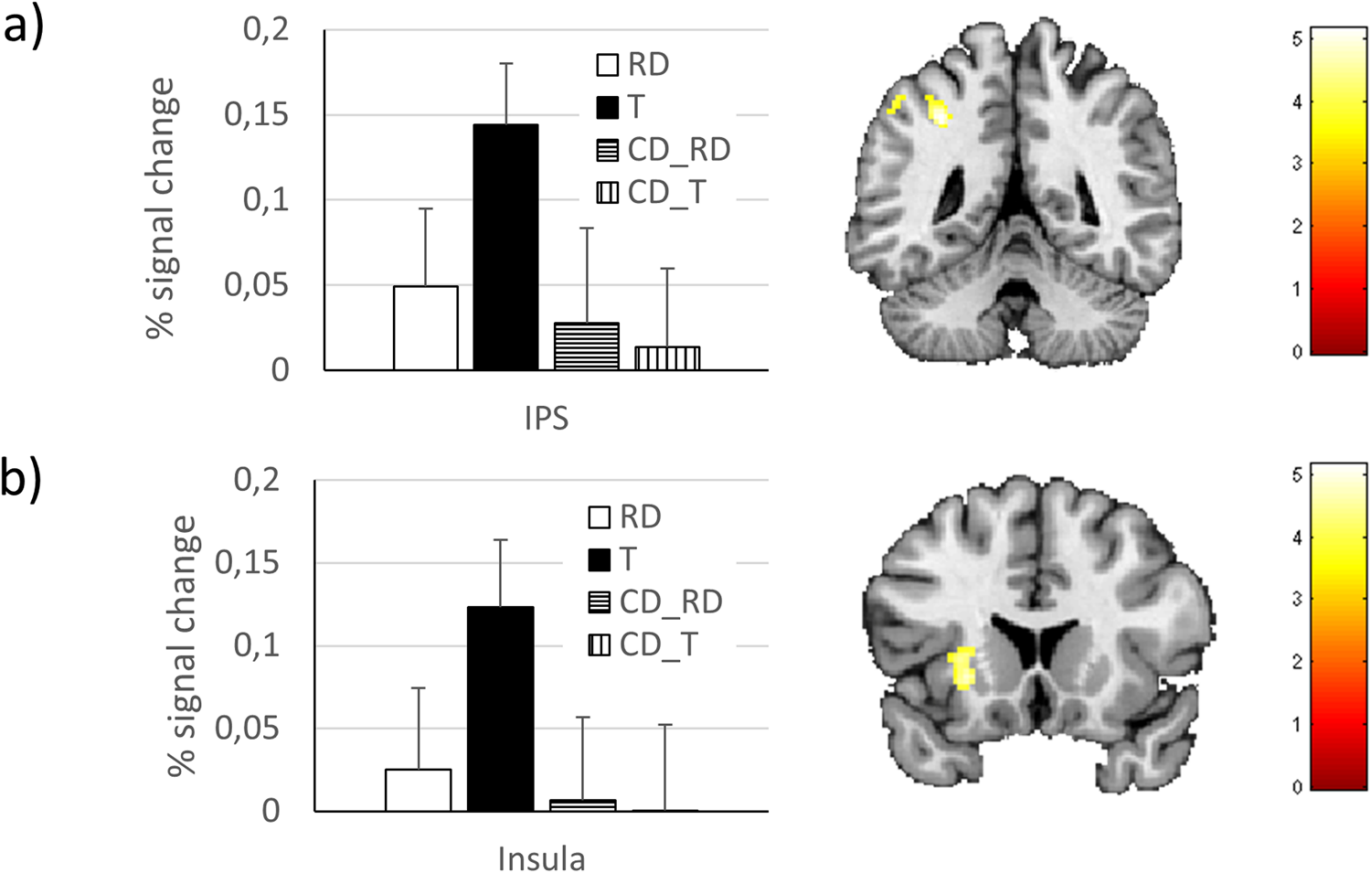
Illustrative ROI-Analysis of the two activated clusters in the contrast targets versus rare distractors (standard GLM analysis): (a) left IPS (b) left insula; threshold:*p*< 0.001 uncorrected, showing only clusters with*p*< 0.05 FWE corrected on cluster level. T = targets, CD_T = common distractors, fixation rank-matched to the targets, RD = rare distractors, CD_RD = common distractors, fixation rank-matched to the rare distractors. Error bars denote the standard error of the mean.

**Fig. 4.**
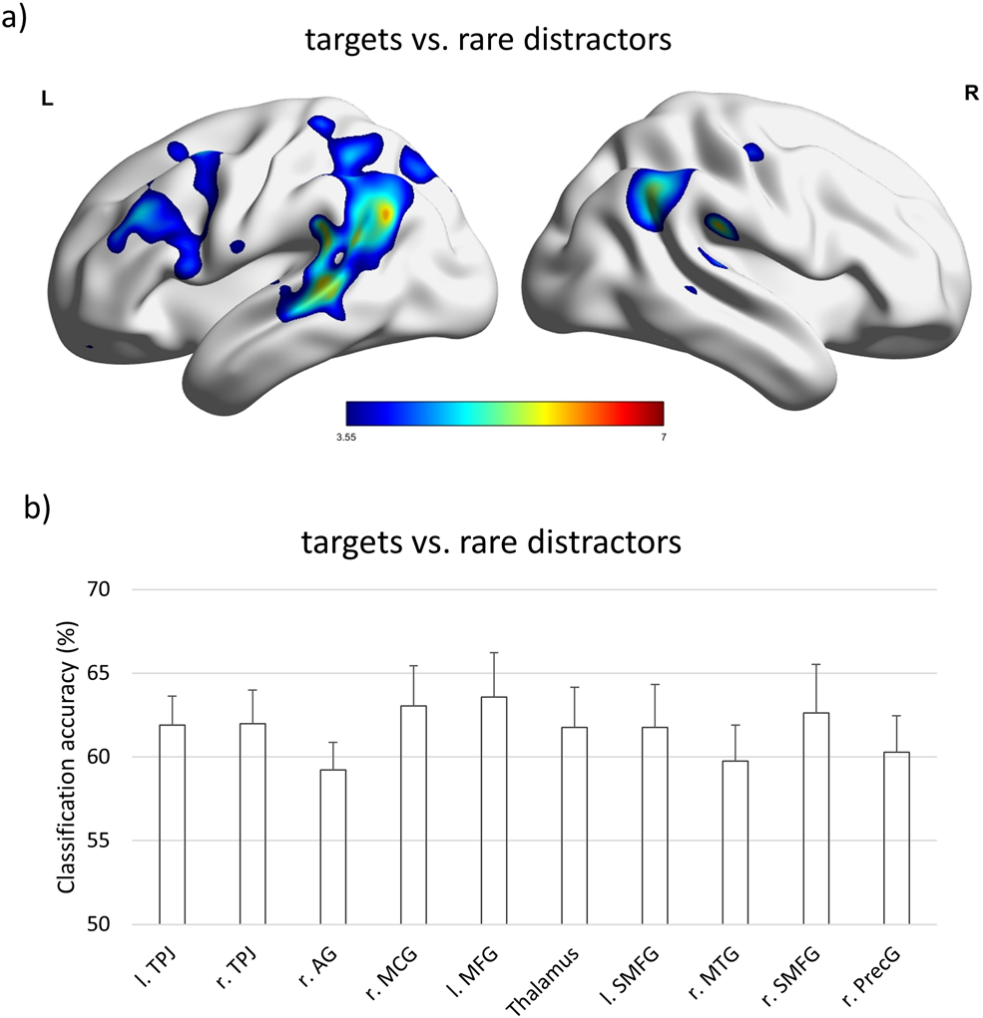
(a) MVPA analysis: T versus RD; threshold:*p*< 0.001 uncorrected, showing only clusters with*p*< 0.05 FWE corrected. Results are visualized with BrainNet Viewer (http://www.nitrc.org/projects/bnv/) byXia et al. (2013). (b) Classification accuracies in the 10 significant clusters (peak voxel). r. = right, l. = left, TPJ = temporo-parietal junction, AG = angular gyrus, MCG = middle cingulate gyrus, SMFG = superior medial frontal gyrus, MFG = middle frontal gyrus, MTG = middle temporal gyrus, PrecG = precentral gyrus. Error bars denote the standard error of the mean.

Second, we investigated brain areas that indicate the processing of task relevance. To investigate which brain areas responded more strongly to targets T than to rare distractors RD, we compared target fixations from the 1T_0RD and 1T_1RD condition with rare distractor fixations from the 0T_1RD and 1T_1RD condition (seeTable 1). We observed more activation for targets than for rare distractors (T > RD) in the left intraparietal sulcus and the left insula (Table 4). We also plotted percent signal change for all four fixation conditions in both ROIs (Fig. 3). No significant activations were observed in the reverse contrast (RD > T). Furthermore, we used MVPA to find out whether local activation patterns differed between targets and rare distractors. The results of this analysis showed that targets and rare distractors could be classified correctly in a wide range of fronto-parietal brain areas, including the TPJ, bilaterally. In the frontal cortex, activation in areas in the left middle frontal gyrus, the right precentral gyrus, and the superior medial frontal gyrus, bilaterally, successfully distinguished targets from rare distractors (Table 5,Fig. 4a). We also extracted the classification accuracies for the peak voxels of all 10 significant cortical clusters (Fig. 4b).

**Table 5.** Results of the MVPA analysis: comparison of target fixations with rare distractor fixations.

Hemisphere	Region	x	y	z	k	Z
	Targets vs. rare distractors					
Left	TPJ	-52	-42	26	6889	4.88
Right	TPJ	56	-28	24	861	4.47
Right	Angular gyrus	58	-56	30	1131	4.27
Right	Middle cingulate gyrus	10	-20	38	1987	4.21
Left	Middle frontal gyrus	-36	26	32	3339	4.04
Left	Thalamus	-14	-8	4	336	3.93
Right	Precentral gyrus	44	-16	52	78	3.80
Left	Superior medial frontal gyrus	-10	30	30	150	3.74
Right	Middle temporal gyrus	50	-40	-4	198	3.72
Right	Superior medial frontal gyrus	4	30	48	180	3.60

*Note.*Statistical parameter maps were thresholded with an initial threshold of*p*< 0.001 uncorrected, reporting only clusters that survived FWE correction on cluster level (*p*< 0.05). Coordinates are reported as given by SPM12 (MNI space). k = cluster size, Z = Z value for the maximally significant voxel of the cluster. Cerebellar areas are not reported due to incomplete coverage of this part of the brain.

An alternative analysis approach is to compare target and rare distractor fixations directly in the 1T_1RD condition. In the GLM analysis, activation in the left insula (-28 18 -2, k = 197, Z = 4.36) was greater for targets than for rare distractors. In the MVPA, four significant clusters distinguished between targets and rare distractors (the left TPJ: -48 -36 32, k = 157, Z = 3.94, the left middle frontal gyrus -32 40 36, k = 231, Z = 3.88, the paracentral lobule -2 -34 52, k = 234, Z = 3.73, and the right Rolandic operculum 34 -38 28, k = 77, Z = 3.54). These results show that the activation differences between targets and rare distractors could be satisfactorily extracted, although these events occurred in close temporal succession within trials of the same condition.

One might argue that some of the differences between targets and rare distractors we had observed stem from comparing targets and rare distractors from partly different conditions (1T_0RD, 1T_1RD for targets and 0T_1RD, 1T_1RD for rare distractors, seeTable 1). To rule out that the observed differences were due to more general differences between conditions, we compared the fixation rank-matched common distractors for targets CD_T with the fixation rank-matched common distractors for rare distractors CD_RD. Both item types were similarly generated but came from partly different conditions. This means that possible condition differences should show up in this contrast. With regard to fixation ranks, both types of distractors were comparable (13.19 and 13.30,*t*(20) = 0.202,*p*= 0.842, ns.). In the GLM analysis as well as the MVPA, no significant differences between both distractor types were obtained.

## 4 Discussion

Using fixation-based event-related analysis, we investigated the processing of task relevance while controlling for item rarity in overt serial visual search. We presented displays that contained targets and/or rare distractors among common distractors. All shared the same visual features and were presented such that they were identifiable only by foveation. Differences in activation between targets and other items could thus not be due to differences induced by peripheral vision. We found that targets and rare distractors activated a number of visual areas as well as areas in the dorsal attention network more strongly than common distractors. To distinguish task relevance from item rarity, we compared fixations on targets with fixations on rare distractors. Two brain areas were more strongly activated for targets than for rare distractors, the left intraparietal sulcus and the left insula. Using MVPA, we also found that, among other areas, activation patterns in the TPJ, bilaterally, distinguished between targets and rare distractors. The intraparietal sulcus is part of the dorsal attention system, whereas the insula and the TPJ are areas usually assigned to the ventral attention system. Our results indicate that these latter areas play a crucial role in the processing of task relevance.

As a first main result, we observed that the processing of targets and rare distractors activated the visual cortex as well as fronto-parietal brain areas comprising the dorsal attention network more strongly than common distractors. These results are very similar to those reported in our previous study (Ischebeck et al., 2021), where we had participants report the number of targets among distractors in the display. With the present experiment we were able to replicate and expand these earlier results.

The dorsal attention network has regularly been reported as being involved in more difficult visual search tasks (Donner et al., 2000,^2002^;Fairhall et al., 2009;Kim et al., 2012;Leonards et al., 2000). According toCorbetta and Shulman (2002), this network is responsible for the top-down volitional deployment of attention required in this task. This is especially true for our demanding multiple-target search task where participants had to correctly identify targets and keep them in working memory throughout the search. However, here we were interested in activations at the moment of fixation of particular items during search. It is less clear whether items such as targets activate the dorsal attention network more than distractors. There is some evidence that the dorsal attention network is involved during target detection.Shulman et al. (2001)have reported that areas associated with the dorsal attention network were more activated during periods in which a coherent motion target embedded in dynamic noise was detected. However, few fMRI studies investigating serial visual search focused specifically on the processing of targets. One possible reason for this is the difference between target present and target absent displays. In searches with one target, participants can abort the search once the target has been found. In target absent trials, participants have to search the display exhaustively. This makes it difficult to interpret activation differences between the two display types as being related to target processing alone. In the present study, we avoided this problem because participants had to exhaustively search the display as long as the display contained less than two targets.

The main objective of the current experiment was to distinguish task relevance from item rarity in serial visual search. To this end, we compared the activation present at the time of target fixation with the activation elicited by rare distractors. It turned out that when target processing was compared to the processing of rare distractors, most activation in the dorsal attention system disappeared. Due to the similar BOLD responses of targets and rare distractors, respectively, in comparison to common distractors, it seems likely that these activations canceled each other out when compared directly. Two brain areas, however, were more strongly activated for targets than for rare distractors, the left intraparietal sulcus and the left insula. Using MVPA, we also found that, among other areas, activation patterns in the TPJ, bilaterally, distinguished between targets and rare distractors. The two analyses pick up different aspects of the BOLD signal. Whereas the standard GLM analysis relies on smoothed data to identify activation differences, the MVPA analysis is conducted on unsmoothed data to identify local differences in activity patterns that distinguish between two conditions. In our experiment, there were only subtle differences between the items: targets and rare distractors. They were equally salient and equally rare or unexpected. If these two properties lead to different activation or deactivation levels within the TPJ, these activations would have vanished in the subtraction of both conditions. It is possible that the activation pattern differences between targets and rare distractors within the TPJ reflect the actual match or mismatch with the internal target template. The higher activation levels of the left IPS and the left insula for targets, on the other hand, might reflect control processes of the dorsal attention system such as memorizing the target and updating a counter.

Activation in the IPS was also observed byMacaluso and Ogawa (2018)who compared the processing of target with distractor fixations in a different search task. In their experiment, participants navigated in search of a target through a virtual environment with differently colored spheres and cubes. Similar to the present study, the authors used a fixation-based analysis to disentangle motor responses from fixation-related activity. They also used different types of distractors, eliminating item rarity as a confounding factor. When participants fixated the target as compared to a distractor, they observed stronger activation in the inferior parietal lobe which was restricted to the right hemisphere. They interpreted this as a result of matching the features of the fixated object, here the target, to the target template. This interpretation fits well the activation in the IPS observed in our study which was also lateralized, albeit to the left hemisphere. It is reasonable to assume that target detection activates the IPS as part of the dorsal attention system. Goal-directed behavior includes the representation of task demands and stimulus-response mappings. In our case, it was necessary to memorize the target in order to give the correct response. The left hemispheric lateralization of the IPS and insula activation observed in our experiment might be related to an interaction of the dorsal attention system with the language system’s involvement in memorizing and updating a counter.

Importantly, we also found that activation patterns in the TPJ, bilaterally, distinguished between targets and rare distractors. This difference might be related to template matching. Each time the gaze falls upon an item in our search displays, it has to be compared to the target template held in working memory. Target recognition in our search task entails the successful matching of the fixated item to the target template. We also found stronger activation of the left anterior insula for target compared to rare distractor fixations. The anterior insula as well as the TPJ have been associated with the detection of task-relevant events in oddball tasks (Bledowski et al., 2004;Kiehl et al., 2005), especially when they are unexpected. TPJ and insula activation were observed to also increase with the length of the target-to-target interval (Fajkus et al., 2015;Stevens et al., 2005) and to decrease with target probability (Horovitz et al., 2002). In our experiment, however, target probability was unlikely to affect the results, as targets and rare distractors were equally unlikely to be encountered during search. This suggests that task relevance is the major explanation for the activation differences within the anterior insula and the TPJ as observed in the present study.

Template matching also plays a role in oddball paradigms. As already outlined in the introduction, the activation on individual rare events in our search paradigm is similar to three-stimuli oddball paradigms where targets and novels are presented occasionally in a stream of numerous similar distractors. The oddball task has been initially studied with EEG (Sutton et al., 1965). In the three-stimuli paradigm, the event-related potentials on targets and novels are the P3b and the P3a, respectively (Comerchero & Polich, 1999). They differ in timing and topography (Polich, 2007;Polich & Comerchero, 2003), indicating that both are generated by a different set of brain areas. FMRI experiments of the three-stimuli oddball task found that predominantly areas of the ventral attention system, of which the TPJ is a part, responded more strongly to targets than to novels (Bledowski et al., 2004;Fajkus et al., 2015;Kiehl et al., 2005). Our results fit well with the results of a meta-analysis of three-stimuli oddball tasks conducted byKim (2014), which yielded, among others, the TPJ and the anterior insula, bilaterally. In the present study, we did not find a significant activation amplitude difference in the TPJ using the univariate analysis. A reason for the lack of an overall amplitude difference in the TPJ might be the visual similarity of the stimuli used in our search task. Whereas novels in three stimuli oddball tasks are perceptually often more salient than targets, our stimuli shared the same features with each other and were equally salient. Different from the results of the GLM, the MVPA analysis yielded differences in local activation patterns between targets and rare distractors, indicating that the TPJ is involved in relevance processing. Based on the view that the TPJ serves a filtering function that is crucial in preventing inappropriate attention shifts (Shulman et al., 2007), such attention shifts might be stronger when distractors are more salient than targets. As differences in TPJ activation (or deactivation) might depend on the relative saliency of targets and rare distractors, this might explain why we did not observe overall activation-level differences in the TPJ.

We have interpreted the TPJ activity as reflecting target relevance. In the influential model byCorbetta and Shulman (2002), the TPJ has been associated with bottom-up reorienting of attention and it is considered a crucial part of the ventral attention system. In this model, especially the right TPJ is assumed to send an early signal that interrupts attentional processing in the dorsal network. It is also assumed that the ventral attention system is predominantly responsible for such stimulus-driven reorienting of attention. It is difficult, however, to attribute the activation of the anterior insula and the results for the TPJ as being due to stimulus-driven attention. All items in our search displays were peripherally equally salient and identifiable only by foveation. This is very different from search tasks, where attention can be guided by a spatial priority map to locations that most likely hold the target (see, e.g.,Wolfe, 2021). The spatial priority map in our search only contained information about locations as saccade targets, with the nearest one being the most likely to be selected next (distance-driven search). This means that the bottom-up flow of information from the spatial priority map would not be affected by a target somewhere in the display. Targets also were not rarer than rare distractors, that is, both were equally unexpected during search. This makes it unlikely that the involvement of the ventral attention system for targets observed in the present study was due to stimulus-driven orienting of attention.

Our interpretation of the TPJ involvement in target processing might be more in line with the idea of later occurring post-perceptual processes such as contextual updating. Contextual updating means that the current cognitive model of events and behavioral consequences is updated due to stimuli that may be behaviorally relevant, such as targets (Donchin & Coles, 1988). In their review on the functional role of the TPJ,Geng and Vossel (2013)point out that TPJ activity involved in relevance processing might not be lateralized. Although some functional hemispheric asymmetries were reported, imaging studies with healthy adults have regularly found a bilateral involvement of the TPJ in attention tasks, for example, in oddball studies (Kiehl et al., 2005;Linden et al., 1999;Stevens et al., 2006) or the spatial cueing paradigm (Jurewicz et al., 2020;Rosen et al., 1999;Vossel et al., 2006,^2009^). To reconcile observations of bilateral versus lateralized TPJ function, it has recently been proposed that the lateralization of TPJ activity might depend on whether there is a match or a mismatch between expected and actual sensory events (Doricchi et al., 2010; for a review, seeDoricchi et al., 2022). In particular, it has been proposed that the left TPJ processes match as well as mismatch signals whereas the right TPJ codes signals mismatch to the attentional template. While we did not observe lateralized activation pattern differences, the MVPA findings are compatible with the view that the TPJs process match and mismatch signals to the attentional template.

Corbetta and Shulman (2002)already assumed that the right TPJ is involved in the processing of item relevance, albeit in a more low-level and bottom-up oriented manner, for example, when promising items appear at unattended locations. The preceding brief discussion of bilateral TPJ activity in coding match and mismatch signals with regard to the attentional template indicates that the TPJs serve a more complex role in attentional processing. Fixating a target item, discriminating it from distractors, matching it to a stored target template, and updating the cognitive model of the environment, accordingly, may be the single most significant instant during an ongoing and demanding attention task like visual search. Our results, therefore, contribute to our knowledge about the function of the TPJ in attention tasks.

## Data Availability

Data and code are available underhttps://openneuro.org/datasets/ds005267
